# Associations between oral microbiome diversity and rheumatoid arthritis in U.S. adults: NHANES 2009–2012

**DOI:** 10.2340/aos.v85.46064

**Published:** 2026-06-26

**Authors:** Jun-Cai Lin, Qi-Song Wang, Zhong-Min Guo, Fan Zhang, Cai-Yin Qiu

**Affiliations:** Department of Stomatology, Longyan First Affiliated Hospital of Fujian Medical University, Longyan, Fujian, China

**Keywords:** Oral microbiome, rheumatoid arthritis, alpha diversity, cross-sectional study, NHANES, Arthritis, Rheumatoid, Microbiota, Mouth, Biodiversity, Nutrition Surveys, Cross-Sectional Studies, Adult

## Abstract

**Objective:**

Although links exist between periodontitis and rheumatoid arthritis (RA), and the gut microbiome has been implicated in RA pathogenesis, the role of oral microbiome diversity in RA remains insufficiently characterized. This study aimed to explore the association between oral microbiome diversity (including alpha and beta diversity) and RA status through a cross-sectional analysis of the National Health and Nutrition Examination Survey (NHANES).

**Material and Methods:**

This cross-sectional study analyzed data from 1,544 participants aged ≥ 20 years derived from the 2009–2012 NHANES cycles. We employed multivariate logistic regression, restricted cubic splines (RCS), receiver operating characteristic (ROC) curve analysis, SHapley Additive exPlanations (SHAP) method and beta diversity assessment (Principal Coordinate Analysis [PCoA] and Permutational Multivariate Analysis of Variance [PERMANOVA]) to examine associations between oral microbiome diversity metrics and RA.

**Results:**

After adjustments, four alpha diversity metrics (observed amplicon sequence variants [ASVs]: OR [95% CI] = 0.996 [0.992, 0.999], *P* = 0.043; Faith’s PD: OR [95% CI] = 0.943 [0.900, 0.988], *P* = 0.014; Shannon-Wiener index: OR [95% CI] = 0.786 [0.641, 0.965], *P* = 0.021; Simpson index: OR [95% CI] = 0.127 [0.018, 0.915], *P* = 0.037) were significantly inversely associated with the presence of RA. This relationship was approximately linear (*P* for nonlinear > 0.05) and moderated by socioeconomic factors (*P* for interaction < 0.05). ROC and SHAP analyses revealed that the Simpson index had the highest explanatory capacity for RA. However, beta diversity (Bray-Curtis, UniFrac distances) revealed no significant differences between RA and non-RA groups (all *P* > 0.05).

**Conclusions:**

Higher oral microbiome alpha diversity is significantly associated with lower prevalence of RA. Oral microbial diversity may serve as a potential indicator associated with RA status. However, given the cross-sectional nature of this study, longitudinal and interventional studies are warranted to further elucidate causal relationships.

## Introduction

Rheumatoid arthritis (RA) is an autoimmune disease that primarily affects the synovial membrane, leading to pain, stiffness, and swelling [1]. Without appropriate treatment, RA can cause severe damage and deformation of joint tissues, potentially resulting in disability, as well as systemic complications such as infections, lymphoma, osteoporosis, and cardiovascular diseases [2]. Furthermore, due to the persistent medical demands and reduced quality of life, RA imposes a significant socioeconomic burden globally [3]. Between 1990 and 2021, there has been a marked global increase in both the age-standardized prevalence rate (ASPR) and the age-standardized incidence rate (ASIR) of RA. In 2021, the global burden of RA was estimated at 17.9 million prevalent cases, 1 million new cases, and 3.08 million disability-adjusted life years (DALYs) [2]. By 2050, the ASIR is projected to rise by 108.7%. Although the exact etiology of RA remains unclear, evidence suggests that both genetic predispositions and environmental factors contribute to its development [4]. Reported risk factors for RA include smoking, obesity, exposure to ultraviolet radiation, sex hormones, medications, alterations in the gut, oral, and lung microbiota, periodontal disease (periodontitis), and infections [1, 5–9].

The human oral microbiome is the second-largest microbial community in the human body, surpassed only by the gut microbiome. It comprises over 700 bacterial species, as well as fungi, viruses, and protozoa [10]. These microorganisms play a critical role in maintaining oral homeostasis by inhibiting the colonization of pathogenic bacteria and promoting the production of salivary antimicrobial components that shape the oral microbiome, such as IgA and defensins [11]. However, when the balance of the bacterial community is disrupted due to factors such as poor oral hygiene, unhealthy diet, smoking, and diabetes, it can lead to both oral diseases and systemic conditions beyond the oral cavity [12]. Substantial evidence indicates that certain species within the oral cavity, such as *Porphyromonas gingivalis*, may contribute to the onset of RA autoimmunity [13–15].

However, there remains a paucity of population-based studies investigating the association between oral microbiome diversity and RA. This study aims to explore the association between oral microbiome diversity (including alpha and beta diversity) and RA status through a cross-sectional analysis of the National Health and Nutrition Examination Survey (NHANES). Notably, the NHANES 2009–2010 and 2011–2012 cycles are the only survey periods that include comprehensive oral microbiome sequencing data (16S rRNA gene sequencing of oral rinse samples), making them uniquely suited for investigating the association between oral microbial diversity and systemic diseases such as RA. No subsequent NHANES cycles have included comparable oral microbiome data, which provides the rationale for utilizing these specific survey periods despite the data being collected over a decade ago [16].

## Methods

### Data source and study participants

The data used in this study were derived from the NHANES, a nationally representative cross-sectional survey conducted by the National Center for Health Statistics (NCHS). Utilizing a stratified multistage probability sampling design, the NHANES assessed the health and nutritional status of non-institutionalized U.S. residents. Data collection incorporated three key approaches: structured interviews conducted in participants’ homes, comprehensive health examinations performed at mobile examination centers, and laboratory analyses of collected biological specimens. All participants provided written informed consent on forms approved by the NCHS Ethics Review Board. The data collection and analysis processes strictly complied with the established research protocols of NHANES.

**Figure 1:**
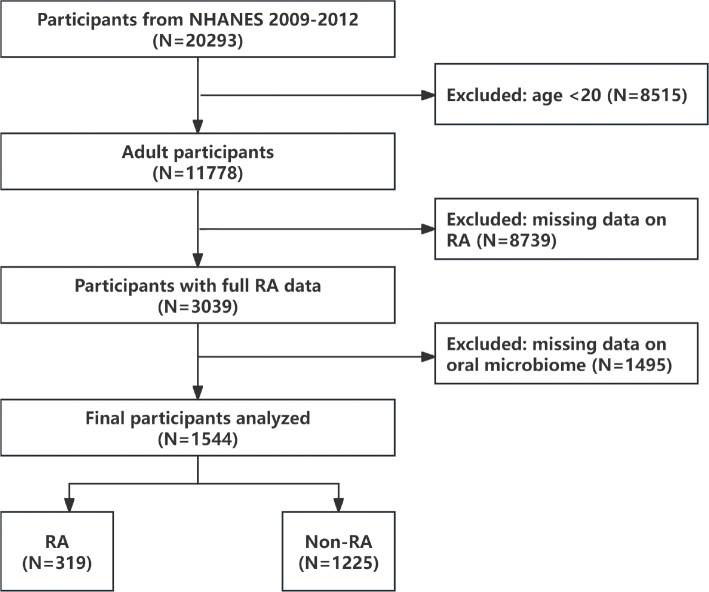
Flowchart of participants’ selection.

Our data were acquired from two NHANES cycles (2009–2010, 2011–2012) based on the availability of oral microbiome data. A total of 1,544 participants aged ≥ 20 years with detailed information on oral microbiome data and RA were enrolled. Figure 1 shows the screening process flowchart.

### Definition of rheumatoid arthritis

The diagnosis of RA was based on self-reported questionnaires. Specifically, participants were initially asked whether they had been diagnosed with arthritis by a physician, and if so, they were required to specify the type of arthritis in detail. A prior study demonstrated a high level of consistency (85%) between self-reported arthritis and clinically confirmed arthritis diagnoses [17]. Nevertheless, it should be acknowledged that self-reported RA in NHANES may still include misclassification with other forms of arthritis, such as osteoarthritis or gout. This potential limitation is further addressed in the Discussion section.

### Oral microbiome diversity

Oral rinse samples collected through NHANES during the periods of 2009–2010 and 2011–2012 were utilized in this study for oral microbiome analysis. Detailed procedures for DNA extraction, sequencing, and bioinformatics analyses were documented on the NHANES website [16]. The results of the oral microbiome testing involved classifying microbial sequences into amplicon sequence variants (ASVs), which were subsequently employed to generate alpha-diversity metrics, beta-diversity metrics, and relative abundance/read count tables across taxonomic levels from phylum to genus [16]. Alpha- and beta-diversity indices describe within-sample and between-sample variations in microbial communities, respectively. Alpha diversity quantifies the diversity of microbial species within a single sample, typically measured by species richness and/or evenness. Beta diversity highlights differences in microbial community composition across ecosystems, such as among individuals [18].

In our study, alpha-diversity was assessed using four indicators: observed ASVs, Faith’s Phylogenetic Diversity (PD), Shannon-Wiener index, and Simpson index. Observed ASVs and Faith’s PD specifically measure species richness, while the Shannon-Wiener and Simpson indices evaluate both richness and evenness. Post-rarefaction data with 10,000 reads per sample were used to compute alpha-diversity metrics, and the mean of 10 replicates was adopted for consistency [16].

Beta-diversity measures included three indices: Bray-Curtis dissimilarity, which evaluates compositional differences between two samples based on species abundance; unweighted UniFrac, which assesses beta diversity based on species presence or absence with an emphasis on rare species; and weighted UniFrac, which integrates phylogenetic relationships and species abundance for a comprehensive understanding of community structure and composition [18].

It is important to note that alpha diversity yields a single continuous value per participant, making it suitable for standard regression modeling (e.g. logistic regression). In contrast, beta diversity is inherently a pairwise distance matrix between all participants rather than a per-individual metric, and thus cannot be directly incorporated into logistic regression models. Instead, beta diversity was analyzed using distance-based multivariate methods, specifically PCoA for visualization and PERMANOVA for statistical testing of group differences [19, 20].

### Covariates

Based on prior literature [21, 22], variables that are either known or hypothesized to be associated with RA, the oral microbiome, or both were selected as covariates for this study: age, gender (male, female), race (Mexican American, Other Hispanic, Non-Hispanic White, Non-Hispanic Black, Other Races), marital status (married/living with partner, never married, widowed/divorced/separated), education level (under high school, high school or equivalent, above high school), poverty income ratio (PIR), body mass index (BMI, kg/m^2^), dietary quality (HEI-2015, Health Eating Index-2015), smoking (never, former, current), drinking (yes, no), hypertension (yes, no), diabetes (yes, no), frequency of dental floss/device usage, and frequency of mouthwash usage.

Dietary quality is assessed using the HEI-2015 score, which incorporates 13 dietary components. Higher scores reflect better dietary quality. Participants with an average HEI-2015 score of 60 or higher were considered to be adhering to the dietary guidelines [23]. Consequently, in subgroup analysis, we categorized dietary quality into two levels: ‘< 60’ or ‘≥ 60’. PIR was classified as ‘< 3.5’ or ‘≥ 3.5’, and age was classified as ‘20–45’ or ‘46–69’ [24] in subgroup analysis.

Smoking and alcohol consumption were assessed through self-reported questionnaires administered during the NHANES home interview, which are susceptible to reporting bias; however, these are widely used measures in large-scale epidemiological studies [25, 26]. Smoking status was categorized as never (< 100 lifetime cigarettes), former (≥ 100 lifetime cigarettes, but quit smoking), or current (≥ 100 lifetime cigarettes and still smoking) [25]. Drinking status was classified as no (< 12 lifetime drinks) or yes (≥ 12 lifetime drinks) [26].

Hypertension was defined as a self-reported prior diagnosis, the use of antihypertensive medication, or a blood pressure reading of ≥ 130/80 mmHg, in accordance with the 2017 American College of Cardiology/American Heart Association (ACC/AHA) hypertension guideline [27].

Diabetes was defined as a self-reported prior diagnosis, or meeting any of the following laboratory criteria: a fasting plasma glucose level of ≥ 7 mmol/L, a glycosylated hemoglobin A1c (HbA1c) level of ≥ 6.5%, a 2-h post-load plasma glucose level of ≥ 11.1 mmol/L, or the use of insulin or oral hypoglycemic medications. These laboratory criteria were applied to confirm diabetes status among participants who did not self-report a prior diagnosis, thereby reducing potential misclassification [28].

Oral hygiene habits, including the frequency of flossing and mouthwash use, were assessed via questionnaire by recording the number of days participants used dental floss/devices and mouthwash during the previous week. The NHANES questionnaire assessed general mouthwash use without specifying the type of product (e.g. antibacterial vs. cosmetic formulations). This lack of detail regarding mouthwash composition represents a limitation, as certain antibacterial mouthwashes containing chlorhexidine or cetylpyridinium chloride may exert differential effects on oral microbial diversity [29]

### Statistical analysis

Continuous variables were presented as mean ± standard deviation (SD) if they followed a normal distribution, or as median (P25, P75) if they deviated from normality. Categorical variables were summarized as counts (percentages). For continuous data analysis, *t*-tests were used for normally distributed datasets, while the Kruskal-Wallis test was applied for non-normally distributed datasets. Categorical data were assessed using chi-square tests, contingent on the data’s appropriateness. Statistical significance was defined as two-sided *P* < 0.05. All statistical analyses were conducted using R software, Version 4.4.2.

We used logistic regression to estimate odds ratios (ORs) and 95% confidence intervals (CIs) for the association between alpha diversity and RA. Two models were fitted: Model 1 was unadjusted, while Model 2 was adjusted for age, gender, race, PIR, BMI, HEI-2015, education, marital status, smoking, drinking, diabetes, hypertension, frequency of dental floss/device usage, and frequency of mouthwash usage. Restricted cubic spline (RCS) analysis with 4 knots placed at the 5th, 35th, 65th, and 95th percentiles of each alpha diversity index was performed to evaluate the potential non-linear or dose-response relationship between alpha diversity and RA.

The association between alpha diversity and RA was evaluated across different covariate subgroups using subgroup analyses, and interaction analyses were also performed. These subgroup and interaction analyses were considered exploratory in nature. Given the multiple comparisons involved, the possibility of type I error should be acknowledged, and these results should be interpreted with caution [30].

The discriminatory capacity and explanatory contribution of the four alpha diversity indices were assessed using receiver operating characteristic (ROC) curve analysis and the Shapley Additive Explanations (SHAP) method, respectively. It should be noted that ROC analysis evaluates discriminatory accuracy (i.e. the ability to correctly classify RA versus non-RA participants), while SHAP values quantify the relative contribution of each feature to the model’s output, representing explanatory importance rather than discriminatory accuracy per se.

The beta diversity differences between RA and non-RA groups were visualized using Principal Coordinate Analysis (PCoA) and statistically tested using Permutational Multivariate Analysis of Variance (PERMANOVA). In the fields of ecology and microbiology, PCoA is a valuable tool for visualizing variations among samples based on given non-Euclidean pairwise distance matrices, such as Bray-Curtis dissimilarity, unweighted UniFrac distance, and weighted UniFrac distance. By projecting observations into lower-dimensional spaces, PCoA facilitates the identification of potential clusters among the samples [31]. PERMANOVA geometrically partitions multivariate variation within the selected dissimilarity measure space according to the specified ANOVA design, employing appropriate distribution-free permutation techniques to derive *P*-values [32]. For covariates with missing values, we performed multiple imputation using the ‘mice’ package in R as part of a sensitivity analysis.

## Results

### Descriptive results

Table 1 displays the baseline characteristics of the study population stratified by RA status. A total of 1,544 participants were enrolled, with a median age of 56 years (P25–P75: 48–62), with females accounting for 59.13% of the study population (*n* = 913). Participants with RA were more likely to be Mexican Americans or non-Hispanic Blacks, have poorer economic status and lower diet quality, attained lower levels of education, and use mouthwash more frequently (all *P* < 0.05). There was no significant difference in gender, age, marital status, smoking, drinking, BMI, diabetes, hypertension, and frequency of dental floss/device usage (all *P* > 0.05).

**Table 1:** Comparison of baseline characteristics according to incidence of RA.

	Overall (*n* = 1,544)	Non-RA (*n* = 1225)	RA (*n* = 319)	*P*
Age, years	56.000 [48.000, 62.000]	56.000 [48.000, 63.000]	56.000 [48.000, 62.000]	0.561
Gender, *n* (%)				0.38
Male	631 (40.868)	508 (41.469)	123 (38.558)	
Female	913 (59.132)	717 (58.531)	196 (61.442)	
Race, *n* (%)				< 0.001
Mexican American	195 (12.630)	144 (11.755)	51 (15.987)	
Other Hispanic	156 (10.104)	127 (10.367)	29 (9.091)	
Non-Hispanic White	676 (43.782)	568 (46.367)	108 (33.856)	
Non-Hispanic Black	429 (27.785)	317 (25.878)	112 (35.110)	
Other Races	88 (5.699)	69 (5.633)	19 (5.956)	
PIR	1.775 [0.940, 3.970]	1.850 [0.970, 4.120]	1.410 [0.890, 3.295]	0.004
BMI, kg/m^2^	30.340 [26.190, 35.460]	30.470 [26.300, 35.400]	29.900 [25.805, 35.675]	0.404
Dietary quality, HEI-2015	50.094 [42.908, 58.751]	50.611 [43.571, 59.171]	48.686 [41.412, 56.985]	0.005
Education, *n* (%)				0.045
Above high school	754 (48.898)	608 (49.714)	146 (45.768)	
High school or equivalent	356 (23.087)	290 (23.712)	66 (20.690)	
Under high school	432 (28.016)	325 (26.574)	107 (33.542)	
Marital status, *n* (%)				0.354
Married/living with partner	876 (56.809)	696 (56.863)	180 (56.604)	
Never married	187 (12.127)	155 (12.663)	32 (10.063)	
Widowed/divorced/separated	479 (31.064)	373 (30.474)	106 (33.333)	
Smoking, *n* (%)				0.91
Current	435 (28.174)	343 (28.000)	92 (28.840)	
Ever	449 (29.080)	355 (28.980)	94 (29.467)	
Never	660 (42.746)	527 (43.020)	133 (41.693)	
Drinking, *n* (%)				0.250
No	394 (26.876)	305 (26.158)	89 (29.667)	
Yes	1072 (73.124)	861 (73.842)	211 (70.333)	
Diabetes, *n* (%)				0.311
No	888 (57.513)	713 (58.204)	175 (54.859)	
Yes	656 (42.487)	512 (41.796)	144 (45.141)	
Hypertension, *n* (%)				0.782
No	545 (35.298)	435 (35.510)	110 (34.483)	
Yes	999 (64.702)	790 (64.490)	209 (65.517)	
Frequency of dental floss/device usage, *n* (%)				0.576
Never	507 (35.356)	398 (34.943)	109 (36.949)	
< 1 per day	484 (33.752)	392 (34.416)	92 (31.186)	
= 1 per day	443 (30.893)	349 (30.641)	94 (31.864)	
Frequency of mouthwash usage, *n* (%)				0.018
Never	566 (39.498)	470 (41.301)	96 (32.542)	
< 1 per day	327 (22.819)	256 (22.496)	71 (24.068)	
= 1 per day	540 (37.683)	412 (36.204)	128 (43.390)	
Alpha diversity				
Observed ASVs	118.746 ± 42.683	119.520 ± 42.018	115.773 ± 45.092	0.163
Faith’s Phylogenetic Diversity	13.663 ± 3.446	13.748 ± 3.394	13.340 ± 3.626	0.060
Shannon-Weiner index	4.525 [4.104, 4.934]	4.537 [4.134, 4.940]	4.457 [3.935, 4.910]	0.022
Simpson Index	0.913 [0.880, 0.935]	0.914 [0.882, 0.936]	0.908 [0.866, 0.932]	0.004

Median [IQR] or mean ± SD for continuous variables and counts (percentage) for categorical variables.

IQR: interquartile range; SD: standard deviation; RA: rheumatoid arthritis; PIR: poverty income ratio; BMI: body mass index; HEI-2015: Health Eating Index-2015; ASVs: amplicon sequence variants.

### Association between four alpha diversity metrics and RA

We employed univariate and multivariate logistic regression models to examine the association between alpha diversity and RA. Table 2 presents the results. In the univariate analysis, observed ASVs (unadjusted OR: 0.998 [0.995, 1.001], *P* = 0.163) and Faith’s PD (unadjusted OR: 0.966 [0.931, 1.001], *P* = 0.060) showed an inverse association with RA, but the association was not significant. However, the Shannon-Wiener index (unadjusted OR: 0.792 [0.671, 0.936], *P* = 0.006) and Simpson index (unadjusted OR: 0.087 [0.017, 0.450], *P* = 0.003) showed a significant negative association with RA. After adjusting for confounding factors, the associations of observed ASVs (adjusted OR: 0.996 [0.992, 0.999], *P* = 0.043), Faith’s PD (adjusted OR: 0.943 [0.900, 0.988], *P* = 0.014), Shannon-Wiener index (adjusted OR: 0.786 [0.641, 0.965], *P* = 0.021), and Simpson index (adjusted OR: 0.127 [0.018, 0.915], *P* = 0.037) were all significant.

**Table 2:** Association between alpha diversity and risk of RA in logistic regression models.

Alpha diversity	Model 1		Model 2	
	OR (95% CI)	*P*-value	OR (95% CI)	*P*-value
Observed ASVs	0.998 (0.995, 1.001)	0.163	**0.996 (0.992, 0.999)**	**0.043**
Faith’s Phylogenetic Diversity	0.966 (0.931, 1.001)	0.060	**0.943 (0.900, 0.988)**	**0.014**
Shannon-Weiner Index	**0.792 (0.671, 0.936)**	**0.006**	**0.786 (0.641, 0.965)**	**0.021**
Simpson Index	**0.087 (0.017, 0.450)**	**0.003**	**0.127 (0.018, 0.915)**	**0.037**

Statistically significant results (p < 0.05) are presented in bold.

Model 1: no covariates were adjusted.

Model 2: adjusted for age, gender, race, PIR, BMI, HEI-2015, education, marital status, smoking, drinking, diabetes, hypertension, frequency of dental floss/device usage, and frequency of mouthwash usage.

OR: odds ratio; CI: confidence interval; RA: rheumatoid arthritis; PIR: poverty income ratio; BMI: body mass index; HEI-2015: Health Eating Index-2015; ASVs: amplicon sequence variants.

The observation that observed ASVs and Faith’s PD became statistically significant only after covariate adjustment suggests the presence of confounding factors that may have masked the underlying associations in the crude analysis. Specifically, covariates such as race, socioeconomic status (PIR), education level, and dietary quality differed significantly between RA and non-RA groups (Table 1). These confounders may have introduced variability that obscured the true associations in the unadjusted models. After controlling for these factors, the negative associations between species richness indices and RA became apparent. This pattern is consistent with the concept of negative confounding, wherein unadjusted estimates are biased toward the null [33].

It is worth noting that, although the adjusted OR for observed ASVs (0.996 per unit increase) was statistically significant, the effect size is extremely small. A one-unit increase in observed ASVs corresponds to only a 0.4% reduction in the odds of RA, which may be of limited clinical significance. The clinical relevance of this finding should therefore be interpreted with caution, particularly in comparison to the larger effect sizes observed for the Shannon-Wiener index (21.4% reduction per unit) and the Simpson index (87.3% reduction per unit) [34].

**Figure 2:**
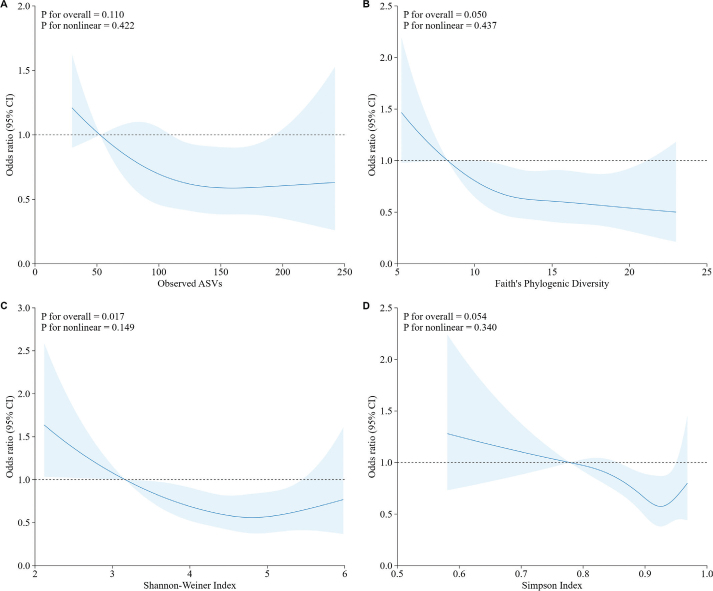
Restrictive cubic spline fitting of the association between alpha diversity metrics and RA. Adjusted for age, gender, race, PIR, BMI, HEI-2015, education, marital status, smoking, drinking, diabetes, hypertension, frequency of dental floss/device usage, and frequency of mouthwash usage. (A) Observed ASVs; (B) Faith’s Phylogenetic Diversity; (C) Shannon-Weiner index; (D) Simpson Index. RA: rheumatoid arthritis; PIR: poverty income ratio; BMI: body mass index; HEI-2015: Health Eating Index-2015; ASVs: amplicon sequence variants.

We examined the dose-response and potential non-linear relationships between various alpha diversity indices and the presence of RA by plotting RCS curves with 4 knots placed at the 5th, 35th, 65th, and 95th percentiles. As shown in Figure 2, the overall tests for nonlinearity were not statistically significant for any of the four alpha diversity indices (all *P* for nonlinear > 0.05), suggesting that the predominant trend is approximately linear. However, visual inspection of the RCS curves reveals some local fluctuations. Specifically, the curve for observed ASVs displays a general downward trend that flattens at higher values (Figure 2A). Faith’s PD shows a pronounced initial decline that gradually attenuates (Figure 2B). The Shannon-Wiener index exhibits an initial decline followed by a modest upward fluctuation at higher values (Figure 2C), and the Simpson index shows a non-monotonic pattern with an initial decrease followed by a slight increase (Figure 2D). These local variations, while not reaching statistical significance for nonlinearity, suggest that the dose-response relationship may not be strictly linear across the full range of diversity values. Therefore, the overall inverse association between alpha diversity and RA should be interpreted as approximately linear, with the caveat that the relationship may deviate from linearity at the extremes of the distribution.

### Subgroup analyses

**Figure 3:**
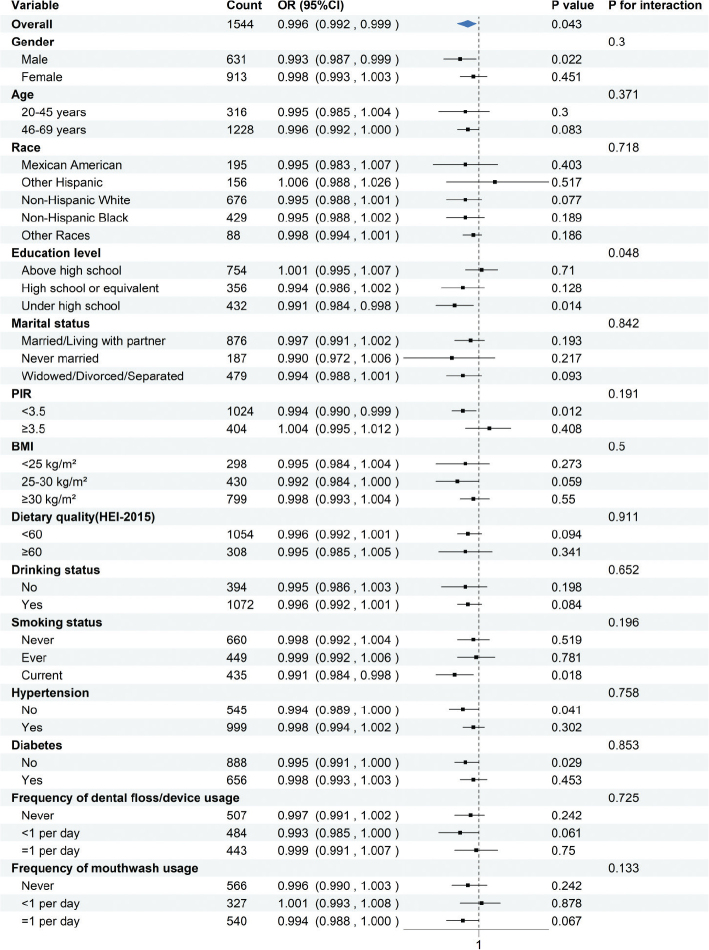
Stratified analyses by potential modifiers of the association between observed ASVs and RA. Adjusted for age, gender, race, PIR, BMI, HEI-2015, education, marital status, smoking, drinking, diabetes, hypertension, frequency of dental floss/device usage, and frequency of mouthwash usage. In the subgroup analysis, the model is not adjusted for the stratification variable itself. OR: odds ratio; CI: confidence interval; RA: rheumatoid arthritis; PIR: poverty income ratio; BMI: body mass index; HEI-2015: Health Eating Index-2015; ASVs: amplicon sequence variants.

Subgroup analyses and interaction tests stratified by various covariates were performed to evaluate the robustness of our findings and to identify potential susceptible populations (Figure 3). As shown in Figure 3, a significant negative association between observed ASVs and RA was observed in male participants, those with low education levels, poor economic conditions, current smokers, and individuals without comorbidities (*P* < 0.05). In the subgroup of participants with poor diet quality (HEI-2015 < 60), the association between observed ASVs and RA showed a trend toward significance, but did not reach statistical significance (*P* = 0.094). The subgroup analysis results of the other three alpha diversity indices were similar (Figures S1–S3). Note: Each supplementary figure has been clearly labeled with its respective designation (Figures S1–S3) in the revised version. Moreover, the results of the interaction test revealed that the *P*-value for interaction within subgroups stratified by education levels was < 0.05, suggesting that education significantly modified the relationship between observed ASVs and RA. For the other three alpha diversity indices (Faith’s PD, Shannon-Wiener index, and Simpson index), we found that PIR significantly moderated their associations with RA (all *P* for interaction < 0.05).

### ROC curve analysis and SHAP analysis

**Figure 4:**
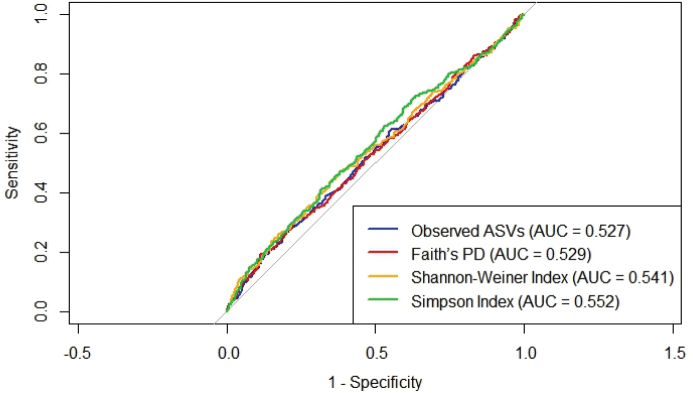
Receiver operating characteristic (ROC) curve analysis. AUC: area under the curve; ASVs: amplicon sequence variants; PD: phylogenetic diversity.

To explore the discriminatory capacity of the four alpha diversity indices for RA, ROC curve analysis was performed. Figure 4 reveals that the Simpson index demonstrated the highest area under the curve (AUC) of 0.552, followed by the Shannon-Wiener index (AUC: 0.541), Faith’s PD (AUC: 0.529), and observed ASVs (AUC: 0.527). It should be noted that these AUC values are only marginally above 0.5 (the threshold for random classification), indicating limited discriminatory ability of individual alpha diversity indices for RA. This finding is not unexpected, as RA is a multifactorial disease influenced by genetic, environmental, and immunological factors, and a single dimension of the oral microbiome would not be expected to serve as a standalone classifier. Table 3 indicates that the Simpson index exhibited the highest specificity (0.624) and Youden index (0.095) but the lowest accuracy (0.498), whereas the observed ASVs demonstrated the highest sensitivity (0.791) and accuracy (0.793) but the lowest Youden index (0.067).

**Table 3:** Performance of models for predicting RA.

Alpha diversity	AUC	Sensitivity	Specificity	Youden Index	Accuracy
Observed ASVs	0.527	0.791	0.276	0.067	0.793
Faith’s PD	0.529	0.886	0.194	0.08	0.793
Shannon-Weiner index	0.541	0.647	0.442	0.089	0.793
Simpson index	0.552	0.471	0.624	0.095	0.498

RA: rheumatoid arthritis; AUC: area under the curve; ASVs: amplicon sequence variants; PD: phylogenetic diversity.

**Figure 5:**
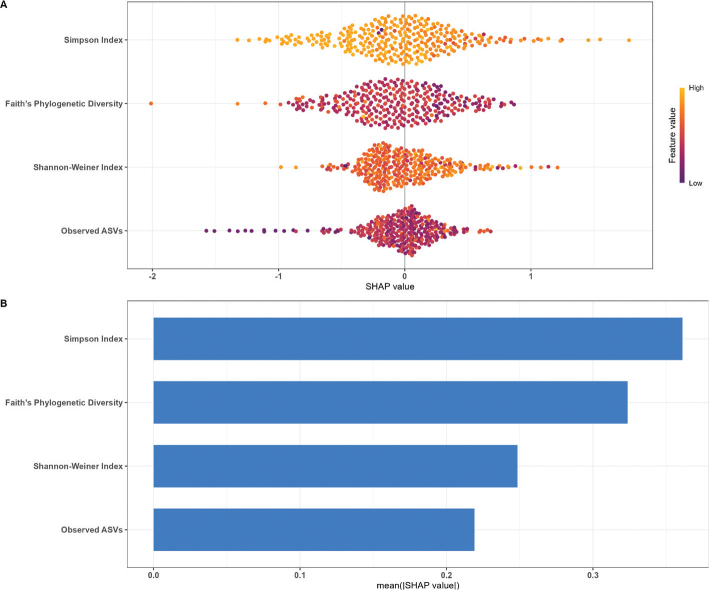
SHAP plot. (A) SHAP beeswarm plot. Yellow dots represent higher eigenvalues and purple dots represent lower eigenvalues. (B) Feature importance ranking as indicated by SHAP.

The relative explanatory contribution of each alpha diversity metric and its contribution to the model’s output was assessed using the SHAP algorithm (Figure 5A and B). The Simpson index had the highest average absolute SHAP value, indicating that it exerts the greatest explanatory influence on the model’s output among the four alpha diversity indices. It is important to distinguish between SHAP-based explanatory importance and AUC-based discriminatory performance: a high SHAP value indicates that a feature contributes substantially to the model’s decision-making process, but this does not necessarily translate into high standalone discriminatory accuracy, as the overall model performance depends on the collective contribution of all features.

We acknowledge that including all four alpha diversity indices simultaneously in the model may introduce multicollinearity, as these indices are inherently correlated measures of microbial diversity. To assess this concern, we calculated the variance inflation factor (VIF) for all four indices in the model, and the results indicated that multicollinearity was within acceptable limits (all VIF < 5). Nevertheless, the correlated nature of these indices should be considered when interpreting the SHAP results.

Using the SHAP beeswarm plot, we found that the lower the feature values of observed ASVs, the greater the positive impact on the model output, while the lower the feature values of Faith’s PD, Shannon-Weiner index, and Simpson index, the greater the negative impact on the model output. These results suggest that the Simpson index exerted the greatest explanatory contribution among the four indices; however, given the limited AUC values (0.527–0.552), the overall discriminatory capacity of these indices as standalone classifiers for RA remains modest. The beeswarm plot should be interpreted as reflecting relative feature importance within the model rather than as evidence of strong classification performance.

### Comparison of oral microbial beta diversity in RA and non-RA populations

**Figure 6:**
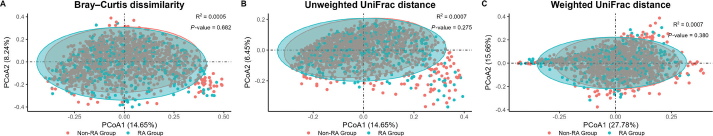
Principal coordinate analysis (PCoA) plots illustrating the differences in oral microbiome beta diversity between RA and non-RA populations. (A) Bray-Curtis dissimilarity; (B) Unweighted UniFrac distance; (C) Weighted UniFrac distance. RA: rheumatoid arthritis.

As illustrated in Figure 6, PCoA analysis was employed to compare the beta diversity metrics between RA patients and non-RA individuals. In order to assess the degree of differences in oral microbial beta diversity between these two groups, a PERMANOVA test was performed. However, no significant differences in these three beta diversity metrics were observed between the RA and non-RA populations (Figure 6A, Bray-Curtis dissimilarity: *R*^2^ = 0.05%; Figure 6B, unweighted UniFrac distance: *R*^2^ = 0.07%; Figure 6C, weighted UniFrac distance: *R*^2^ = 0.07%; all *P* > 0.05).

### Sensitivity analysis

The missingness of the covariates in this analysis is summarized in Table S1. Among these, PIR, BMI, drinking, education, marital status, frequency of dental floss/device usage, frequency of mouthwash usage, and HEI-2015 exhibited missing values. Notably, the missing proportions were relatively low, with all variables except HEI-2015 (11.79%) having missing rates below 10%. The results of the baseline analysis performed using the imputed dataset (Table S2) were consistent with those obtained from the unimputed dataset (Table 1). Similarly, consistency was also observed in the logistic analysis results (Table S3). Thus, it can be concluded that the presence of missing values in the covariates does not affect the primary analysis results of this study.

## Discussion

This study found that, after adjusting for all relevant covariates, alpha diversity (observed ASVs, Faith’s PD, Shannon-Weiner index, Simpson index) was negatively associated with the presence of RA, with higher alpha diversity associated with lower prevalence of RA. The RCS analysis results further confirmed an approximately linear inverse association between alpha diversity and RA status, though modest local fluctuations were noted in the dose-response curves. Education level and PIR respectively moderated the associations of observed ASVs and the other three alpha diversity indices with RA. Among the four alpha diversity indices, the Simpson index exhibited the greatest explanatory contribution to the model, although its discriminatory capacity as a standalone classifier remained limited (AUC = 0.552). Sensitivity analysis results validated the robustness of these findings. Finally, beta diversity metrics, including Bray-Curtis dissimilarity, unweighted UniFrac distance, and weighted UniFrac distance, revealed no significant differences in oral microbiota diversity between the RA group and the non-RA group.

Among the four alpha diversity indices employed, observed ASVs and Faith’s PD quantify species richness (the number of distinct taxa), whereas the Shannon-Wiener and Simpson indices additionally incorporate species evenness (the uniformity of taxa distribution). This study revealed that higher alpha diversity was significantly associated with a lower prevalence of RA, particularly for the Shannon-Weiner index and Simpson index. Specifically, a one-unit increase in observed ASVs, Faith’s PD, Shannon-Weiner index, and Simpson index corresponded to a 0.4%, 5.7%, 21.4%, and 87.3% reduction in the odds of RA, respectively. Although the effect size for observed ASVs (OR = 0.996) is statistically significant, its clinical relevance is minimal given the extremely small magnitude. In contrast, the Shannon-Wiener and Simpson indices demonstrated substantially larger effect sizes, suggesting that both species richness and evenness may be more meaningful ecological dimensions in the context of RA. This finding implies that the association between oral microbial diversity and RA may be more closely related to the overall ecological balance of the microbial community (encompassing both richness and evenness) rather than species count alone.

Currently, there is no consistent conclusion regarding the association between the alpha diversity of the oral microbiome and RA. A prior meta-analysis demonstrated that, compared with healthy controls, the alpha diversity of the oral microbiome in RA patients either increased or remained stable, whereas a decrease or stability in the alpha diversity of the gut microbiome was more prevalent among RA patients [35]. Importantly, the present study found that RA participants exhibited lower alpha diversity compared with non-RA participants (though differences were statistically significant only for the Shannon-Wiener index and Simpson index in the unadjusted analysis). After adjusting for confounding factors, all four alpha diversity metrics showed significant inverse associations with RA. This apparent discrepancy with prior studies reporting increased oral alpha diversity in RA patients may be attributable to several methodological differences: (1) population characteristics – our study utilized a large, nationally representative U.S. cohort, whereas prior studies were typically conducted in smaller clinical samples from specific geographic regions; (2) sample collection methods – NHANES employed oral rinse samples, which capture a broader representation of the oral microbial community compared with subgingival plaque samples commonly used in prior studies; and (3) RA ascertainment – our study relied on self-reported diagnosis, potentially including participants across a wider spectrum of disease severity compared with clinically verified cohorts.

Another study reported that in ‘pre-clinical’ high-risk individuals, microbial diversity and richness were significantly lower than in both established RA patients and healthy controls [36]. It is important to note that this finding pertains specifically to a comparison between pre-clinical individuals at elevated risk of RA and those with established disease, which represents a different comparative framework from the present study (RA vs. non-RA). Nevertheless, both studies converge on the observation that reduced diversity may be associated with certain stages or aspects of RA pathogenesis. Additionally, Holers et al. reported an increased relative abundance of potentially pro-inflammatory species in the oral microbiota of RA patients [37]. However, that study focused primarily on compositional differences (beta diversity and relative abundance of specific taxa) rather than on alpha diversity per se; therefore, direct comparison with our alpha diversity findings should be made with caution, as these represent conceptually distinct ecological dimensions.

The reduction of microbial diversity is the main feature of dysbiosis [38]. A simultaneous decrease in microbial diversity coupled with the expansion of specific bacterial taxa may drive the transition of natural oral microbiota dynamics toward ecological dysbiosis. The potential mechanism linking oral microbiota dysbiosis to RA is the induction of systemic inflammation. The presence of autoantibodies, such as rheumatoid factor (RF) and anti-citrullinated peptide antibodies (ACPA), serves as a critical indicator of RA [39]. *Porphyromonas gingivalis*, a periodontal pathogen, is capable of producing peptidylarginine deiminase (PAD) enzymes, which catalyze the citrullination of antigens [36]. The detection of *P. gingivalis*-specific antibodies has been shown to be positively correlated with elevated ACPA titers [40].

While the present study did not assess specific microbial taxa or compositional changes, the observed inverse association between alpha diversity and RA is consistent with the hypothesis that dysbiosis – characterized by reduced diversity and potential expansion of pathogenic taxa such as *P. gingivalis* – may contribute to the inflammatory processes underlying RA. Future studies integrating both diversity metrics and species-level compositional analyses in longitudinal designs would help clarify the mechanistic pathways linking oral dysbiosis to RA.

Furthermore, oral bacteria may contribute to RA via the ‘oral-gut-joint axis’ [10, 12, 41]. Specifically, oral microbiota can supply the gut with colitogenic bacteria and pathogenic Th17 cells, which can lead to colitis, increased gut permeability, and the translocation of additional bacteria across the compromised gut barrier [42]. Subsequently, the gut microbiota may drive excessive activation of both innate and adaptive immune responses within local tissues, contributing to systemic immune dysregulation [43]. Furthermore, alterations in the gut microbiota composition can promote the migration of autoreactive cells to the joints, thereby inducing localized joint inflammation [44]. Two lines of evidence provide support for this association. In mice, gut barrier dysfunction occurs prior to the clinical onset of arthritis [45]. In humans, serum markers indicative of impaired gut barrier function, such as zonulin, are elevated before the development of RA [45]. Both animal and human studies have demonstrated that RA is closely associated with compromised gut epithelial barrier function. Although our study cannot directly test this mechanistic pathway, the observed association between reduced oral microbial diversity and RA is consistent with the theoretical framework of the oral–gut–joint axis, providing a rationale for future mechanistic and interventional investigations.

It is worth noting that the subgroup analysis highlighted the moderating effects of educational level and PIR on the association between oral microbiota and RA. Rather than attributing this effect modification to specific covariates such as dietary habits – whose interaction terms with alpha diversity were not statistically significant – it may be more appropriate to conceptualize education and PIR as upstream social determinants that capture a broader constellation of behavioral and socioeconomic factors [46]. Individuals with different educational and economic backgrounds may differ in a wide range of health-related exposures, including cumulative inflammatory burden, chronic psychosocial stress, early-life environmental exposures, healthcare access and utilization, and health literacy [47]. These factors could collectively modulate the relationship between oral microbial diversity and systemic inflammation, thereby influencing the observed association with RA. Additionally, socioeconomic status may influence oral hygiene behaviors, which are important determinants of oral microbiome composition. For instance, excessive use of antimicrobial mouthwashes has been shown to markedly reduce oral microbial diversity [48], potentially disrupting ecological homeostasis. Thus, the observed effect modification by education and PIR likely reflects the cumulative influence of multiple interconnected socioeconomic pathways rather than any single behavioral factor.

The clinical implications of these findings should be interpreted with appropriate caution given the cross-sectional study design. Specifically, it is not possible to determine from the present data whether reduced oral microbiome diversity precedes RA onset or represents a consequence of the disease or its treatment. Therefore, rather than framing oral microbial diversity as a tool to identify individuals at ‘high risk’ for RA, it is more appropriate to describe it as a factor associated with RA status. Nevertheless, the observed association raises the hypothesis that strategies aimed at maintaining or enhancing oral microbial alpha diversity – such as optimized oral hygiene practices and dietary modifications – may warrant investigation in longitudinal and interventional studies as potential components of RA prevention strategies. Furthermore, the observed effect modification by socioeconomic factors underscores the need for health education programs that are tailored to diverse population subgroups.

## Limitations

Although this study has yielded important insights, it is essential to acknowledge certain limitations. First, the cross-sectional design precluded determining whether changes in oral microbiome diversity are a cause or consequence of RA, thereby limiting causal inference. Second, RA itself and its treatments (e.g. disease-modifying antirheumatic drugs [DMARDs], biologic agents, and corticosteroids) may directly influence the oral environment and microbiome composition through immunosuppressive and anti-inflammatory effects, raising the possibility of reverse causation. This represents an important confound that cannot be resolved without longitudinal data [49]. Third, due to data constraints inherent to NHANES, specific comparisons of oral microbial compositions at the species level were not feasible. Fourth, while several potential confounding factors were accounted for, residual confounding remained possible. For example, family history, which is strongly associated with RA, was unavailable in the NHANES database, potentially introducing bias into the results. Fifth, RA diagnosis in this study relied on self-reported data without objective validation, which may introduce information bias. Although a prior study reported approximately 85% agreement between self-reported and clinically confirmed arthritis diagnoses [50], self-reported RA in NHANES may still include misclassification with other forms of arthritis, such as osteoarthritis or gout, which could attenuate or bias the observed associations. Sixth, the individual alpha diversity indices demonstrated limited discriminatory ability for RA classification (AUC: 0.527–0.552), which is only marginally above random chance. This underscores that oral microbial diversity alone is insufficient for the clinical discrimination of RA and should be considered as one component within a multifactorial risk framework. Seventh, the NHANES data used in this study were collected between 2009 and 2012, and temporal changes in population characteristics, dietary patterns, and healthcare practices may limit the generalizability of these findings to contemporary populations. Despite these limitations, our findings provide valuable insights for future investigations into the role of the oral microbiome in RA. Future research should involve collecting more comprehensive longitudinal data to elucidate the temporal relationship and potential bidirectional interactions between RA and oral microbiome diversity.

## Conclusion

In conclusion, our findings indicate a significant association between oral microbiome alpha diversity and RA status. Specifically, greater oral microbiome alpha diversity is significantly inversely associated with the prevalence of RA, and this association is moderated by educational level and economic status. However, due to the inherent limitations of the cross-sectional design, no inferences regarding causality or temporal directionality can be drawn from these findings. It remains unclear whether reduced oral microbial diversity precedes RA development or results from the disease and its treatment. Future longitudinal and interventional studies are warranted to establish the temporal sequence and to evaluate whether strategies aimed at maintaining oral microbial diversity may contribute to RA prevention or management.

## Supplementary Material

Associations between oral microbiome diversity and rheumatoid arthritis in U.S. adults: NHANES 2009–2012

Associations between oral microbiome diversity and rheumatoid arthritis in U.S. adults: NHANES 2009–2012

## Declarations

### Availability of data and materials

The datasets generated and/or analyzed during the current study are available from the corresponding author on reasonable request.
