# Supplementary material for: Associations between oral microbiome diversity and rheumatoid arthritis in U.S. adults: NHANES 2009–2012
Source: Acta Odontol Scand. 2026 Jun 26;85:46064. doi: 10.2340/aos.v85.46064 (PMC13316869; doi:10.2340/aos.v85.46064)
Supplement: Associations between oral microbiome diversity and rheumatoid arthritis in U.S. adults: NHANES 2009–2012 [file AOS-85-46064-s1.pdf]

Supplementary material has been published as submitted. It has not been copyedited or typeset by Acta Odontologica Scandinavica.

Table S1 The count and percent of missing data in this analysis

Variables	NA_Count	NA_Percent
Gender	0	0
Age	0	0
Race	0	0
PIR	116	7.51
BMI	17	1.1
smoking	0	0
drinking	78	5.05
Education	2	0.13
Marital status	2	0.13
diabetes	0	0
Hypertension	0	0
Frequency of dental floss/device usage, n (%)	110	7.12
Frequency of mouthwash usage, n (%)	111	7.19
HEI-2015	182	11.79
RA	0	0
Observed ASVs	0	0
Faith’s Phylogenetic Diversity	0	0
Shannon-Weiner index	0	0
Simpson Index	0	0

Abbreviations: RA, rheumatoid arthritis; PIR, poverty income ratio; BMI, body mass index; HEI-2015, Health Eating Index-2015; ASVs, amplicon sequence variants.

Table S2 Comparison of baseline characteristics after imputation

	Overall (n=1544)	Non-RA (n=1225)	RA (n=319)	P
Age, years	56.000 [48.000, 62.000]	56.000 [48.000, 63.000]	56.000 [48.000, 62.000]	0.561
Gender, n (%)				0.38
Male	631 (40.868)	508 (41.469)	123 (38.558)	
Female	913 (59.132)	717 (58.531)	196 (61.442)	
Race, n (%)				<0.001
Mexican American	195 (12.630)	144 (11.755)	51 (15.987)	
Other Hispanic	156 (10.104)	127 (10.367)	29 (9.091)	
Non-Hispanic White	676 (43.782)	568 (46.367)	108 (33.856)	
Non-Hispanic Black	429 (27.785)	317 (25.878)	112 (35.110)	
Other Races	88 (5.699)	69 (5.633)	19 (5.956)	
PIR	1.760 [0.940, 4.015]	1.850 [0.950, 4.130]	1.530 [0.910, 3.400]	0.017
BMI, kg/m^2	30.380 [26.190, 35.483]	30.500 [26.300, 35.440]	29.910 [25.880, 35.675]	0.412
Dietary quality, HEI-2015	49.951 [42.900, 58.517]	50.331 [43.558, 58.939]	48.717 [41.366, 57.199]	0.011
Education, n (%)				0.044
Above high school	756 (48.964)	610 (49.796)	146 (45.768)	
High school or equivalent	356 (23.057)	290 (23.673)	66 (20.690)	
Under high school	432 (27.979)	325 (26.531)	107 (33.542)	
Marital status, n (%)				0.403
Married/Living with partner	876 (56.736)	696 (56.816)	180 (56.426)	
Never married	189 (12.241)	156 (12.735)	33 (10.345)	
Widowed/Divorced/Separated	479 (31.023)	373 (30.449)	106 (33.229)	
Smoking, n (%)				0.910
Current	435 (28.174)	343 (28.000)	92 (28.840)	
Ever	449 (29.080)	355 (28.980)	94 (29.467)	
Never	660 (42.746)	527 (43.020)	133 (41.693)	
Drinking, n (%)				0.203

No	414 (26.813)	319 (26.041)	95 (29.781)	
Yes	1130 (73.187)	906 (73.959)	224 (70.219)	
Diabetes, n (%)				0.023
No	2302 (76.478)	2028 (77.169)	274 (71.728)	
Yes	708 (23.522)	600 (22.831)	108 (28.272)	
Hypertension, n (%)				0.003
No	1764 (58.605)	1567 (59.627)	197 (51.571)	
Yes	1246 (41.395)	1061 (40.373)	185 (48.429)	
Frequency of dental floss/device usage, n (%)				0.439
Never	545 (35.298)	427 (34.857)	118 (36.991)	
<1 per day	526 (34.067)	427 (34.857)	99 (31.034)	
=1 per day	473 (30.635)	371 (30.286)	102 (31.975)	
Frequency of mouthwash usage, n (%)				0.012
Never	612 (39.637)	507 (41.388)	105 (32.915)	
<1 per day	356 (23.057)	281 (22.939)	75 (23.511)	
=1 per day	576 (37.306)	437 (35.673)	139 (43.574)	
Alpha diversity				
Observed ASVs	118.746±42.683	119.520±42.018	115.773±45.092	0.163
Faith's Phylogenetic Diversity	13.663±3.446	13.748±3.394	13.340±3.626	0.060
Shannon-Weiner index	4.525 [4.104, 4.934]	4.537 [4.134, 4.940]	4.457 [3.935, 4.910]	0.022
Simpson Index	0.913 [0.880, 0.935]	0.914 [0.882, 0.936]	0.908 [0.866, 0.932]	0.004

Median [IQR] or mean ± SD for continuous variables and counts (percentage) for categorical variables. Abbreviations: IQR, interquartile range; SD, standard deviation; RA, rheumatoid arthritis; PIR, poverty income ratio; BMI, body mass index; HEI-2015, Health Eating Index-2015; ASVs, amplicon sequence variants.

Table S3 Association between alpha diversity and risk of RA using imputed data

Alpha diversity	Model 1		Model 2	
	OR (95%CI)	*P*-value	OR (95%CI)	*P*-value
Observed ASVs	0.998 (0.995, 1.001)	0.163	**0.997 (0.994, 0.999)**	**0.047**
Faith's Phylogenetic Diversity	0.966 (0.931, 1.001)	0.060	**0.954 (0.918, 0.991)**	**0.015**
Shannon-Weiner index	**0.792 (0.671, 0.936)**	**0.006**	**0.781 (0.656, 0.929)**	**0.005**
Simpson Index	**0.087 (0.017, 0.444)**	**0.003**	**0.086 (0.016, 0.472)**	**0.005**

Model 1: no covariates were adjusted.

Model 2: adjusted for age, gender, race, PIR, BMI, HEI-2015, education, marital status, smoking, drinking, diabetes, hypertension, frequency of dental floss/device usage, and frequency of mouthwash usage.

Abbreviations: OR, odds ratio; CI, confidence interval; RA, rheumatoid arthritis; PIR, poverty income ratio; BMI, body mass index; HEI-2015, Health Eating Index-2015; ASVs, amplicon sequence variants.
