# Supplementary material for: Associations between oral microbiome diversity and rheumatoid arthritis in U.S. adults: NHANES 2009–2012
Source: Acta Odontol Scand. 2026 Jun 26;85:46064. doi: 10.2340/aos.v85.46064 (PMC13316869; doi:10.2340/aos.v85.46064)

Supplementary material has been published as submitted. It has not been copyedited or typeset by Acta Odontologica Scandinavica.

Supplementary figure legends

Figure S1 Stratified analyses by potential modifiers of the association between Faith's Phylogenetic Diversity and RA. Adjusted for age, gender, race, PIR, BMI, HEI-2015, education, marital status, smoking, drinking, diabetes, hypertension, frequency ofdental floss/device usage, and frequency of mouthwash usage. In the subgroup analysis, the model is not adjusted for the stratification variable itself. Abbreviations: OR, odds ratio; CI, confidence interval; RA, rheumatoid arthritis; PIR, poverty income ratio; BMI, body mass index; HEI-2015, Health Eating Index-2015.

Variable	Count	OR (95% CI)	P value	P for interaction
**Overall**	1544	0.943 (0.900, 0.988)	0.014	
**Gender**				0.396
Male	631	0.909 (0.841, 0.980)	0.014	
Female	913	0.959 (0.902, 1.019)	0.18	
**Age**				0.428
20-45 years	316	0.936 (0.827, 1.054)	0.281	
46-69 years	1228	0.944 (0.896, 0.994)	0.029	
**Race**				0.719
Mexican American	195	0.919 (0.778, 1.074)	0.299	
Other Hispanic	156	1.092 (0.872, 1.398)	0.459	
Non-Hispanic White	676	0.924 (0.858, 0.993)	0.034	
Non-Hispanic Black	429	0.940 (0.858, 1.027)	0.173	
Other Races	88	0.960 (0.918, 1.004)	0.076	
**Education level**				0.052
Above high school	754	1.004 (0.933, 1.080)	0.914	
High school or equivalent	356	0.910 (0.820, 1.006)	0.072	
Under high school	432	0.891 (0.814, 0.971)	0.01	
**Marital status**				0.789
Married/Living with partner	876	0.958 (0.900, 1.020)	0.181	
Never married	187	0.858 (0.685, 1.052)	0.155	
Widowed/Divorced/Separated	479	0.914 (0.840, 0.991)	0.032	
**PIR**				0.043
<3.5	1024	0.923 (0.874, 0.973)	0.003	
≥3.5	404	1.035 (0.932, 1.147)	0.518	
**BMI**				0.439
<25 kg/m^2	298	0.929 (0.822, 1.042)	0.223	
25-30 kg/m^2	430	0.897 (0.810, 0.990)	0.033	
≥30 kg/m^2	799	0.975 (0.915, 1.038)	0.433	
**Dietary quality(HEI-2015)**				0.987
<60	1054	0.946 (0.898, 0.995)	0.031	
≥60	308	0.937 (0.828, 1.057)	0.295	
**Drinking status**				0.721
No	394	0.929 (0.842, 1.021)	0.128	
Yes	1072	0.943 (0.893, 0.995)	0.034	
**Smoking status**				0.146
Never	660	0.958 (0.886, 1.034)	0.275	
Ever	449	0.990 (0.906, 1.082)	0.826	
Current	435	0.885 (0.809, 0.964)	0.006	
**Hypertension**				0.683
No	545	0.927 (0.865, 0.992)	0.029	
Yes	999	0.966 (0.921, 1.012)	0.146	
**Diabetes**				0.88
No	888	0.934 (0.886, 0.984)	0.011	
Yes	656	0.969 (0.914, 1.026)	0.282	
**Frequency of dental floss/device usage**				0.905
Never	507	0.947 (0.881, 1.018)	0.143	
<1 per day	484	0.915 (0.833, 1.002)	0.059	
=1 per day	443	0.958 (0.870, 1.054)	0.381	
**Frequency of mouthwash usage**				0.277
Never	566	0.935 (0.861, 1.012)	0.099	
<1 per day	327	0.987 (0.896, 1.085)	0.783	
=1 per day	540	0.935 (0.864, 1.009)	0.087	

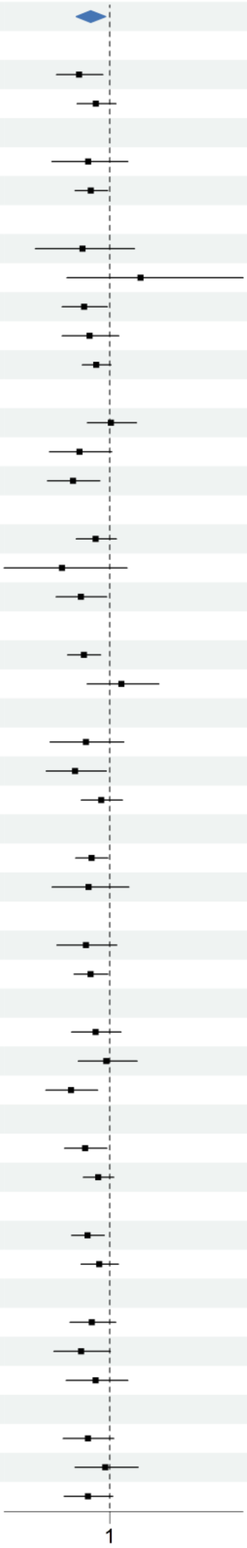

Figure S2 Stratified analyses by potential modifiers of the association between Shannon-Weiner Index and RA. Adjusted for age, gender, race, PIR, BMI, HEI-2015, education, marital status, smoking, drinking, diabetes, hypertension, frequency of dental floss/device usage, and frequency of mouthwash usage. In the subgroup analysis, the model is not adjusted for the stratification variable itself. Abbreviations: OR, odds ratio; CI, confidence interval; RA, rheumatoid arthritis; PIR, poverty income ratio; BMI, body mass index; HEI-2015, Health Eating Index-2015.

Variable	Count	OR (95% CI)	P value	P for interaction
**Overall**	1544	0.786 (0.641, 0.965)	0.021	
**Gender**				0.137
Male	631	0.574 (0.395, 0.831)	0.003	
Female	913	0.888 (0.688, 1.152)	0.367	
**Age**				0.466
20-45 years	316	0.804 (0.516, 1.251)	0.328	
46-69 years	1228	0.786 (0.617, 1.002)	0.051	
**Race**				0.777
Mexican American	195	1.022 (0.483, 2.186)	0.955	
Other Hispanic	156	0.957 (0.286, 3.353)	0.944	
Non-Hispanic White	676	0.733 (0.541, 0.996)	0.045	
Non-Hispanic Black	429	0.691 (0.456, 1.042)	0.078	
Other Races	88	0.834 (0.683, 1.020)	0.076	
**Education level**				0.051
Above high school	754	0.986 (0.720, 1.368)	0.93	
High school or equivalent	356	0.730 (0.454, 1.182)	0.195	
Under high school	432	0.559 (0.374, 0.821)	0.004	
**Marital status**				0.828
Married/Living with partner	876	0.780 (0.602, 1.011)	0.059	
Never married	187	0.668 (0.243, 1.760)	0.418	
Widowed/Divorced/Separated	479	0.781 (0.524, 1.165)	0.222	
**PIR**				0.012
<3.5	1024	0.706 (0.561, 0.887)	0.003	
≥3.5	404	1.371 (0.824, 2.329)	0.233	
**BMI**				0.229
<25 kg/m²	298	0.762 (0.446, 1.294)	0.313	
25-30 kg/m²	430	0.615 (0.403, 0.934)	0.022	
≥30 kg/m²	799	0.940 (0.701, 1.267)	0.683	
**Dietary quality(HEI-2015)**				0.309
<60	1054	0.822 (0.657, 1.032)	0.089	
≥60	308	0.541 (0.314, 0.924)	0.024	
**Drinking status**				0.284
No	394	0.966 (0.638, 1.487)	0.874	
Yes	1072	0.714 (0.559, 0.912)	0.007	
**Smoking status**				0.661
Never	660	0.794 (0.569, 1.111)	0.174	
Ever	449	0.901 (0.590, 1.382)	0.63	
Current	435	0.701 (0.476, 1.027)	0.068	
**Hypertension**				0.998
No	545	0.758 (0.564, 1.019)	0.065	
Yes	999	0.783 (0.629, 0.978)	0.03	
**Diabetes**				0.361
No	888	0.701 (0.553, 0.887)	0.003	
Yes	656	0.864 (0.660, 1.135)	0.287	
**Frequency of dental floss/device usage**				0.679
Never	507	0.789 (0.574, 1.082)	0.14	
<1 per day	484	0.672 (0.444, 1.019)	0.059	
=1 per day	443	0.909 (0.605, 1.388)	0.652	
**Frequency of mouthwash usage**				0.713
Never	566	0.748 (0.538, 1.045)	0.085	
<1 per day	327	0.846 (0.554, 1.295)	0.436	
=1 per day	540	0.814 (0.563, 1.178)	0.274	

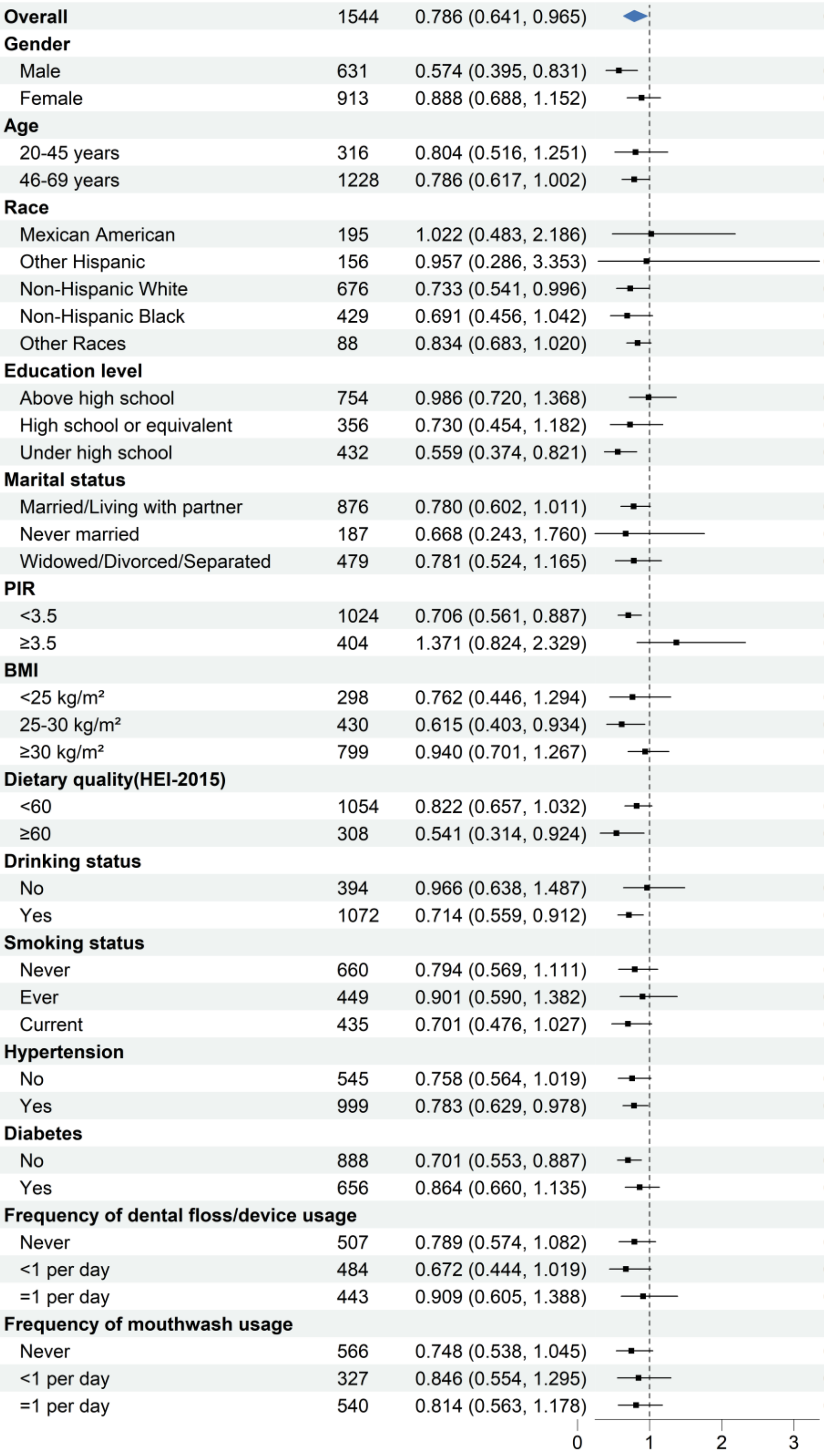

Figure S3 Stratified analyses by potential modifiers of the association between Simpson Index and RA. Adjusted for age, gender, race, PIR, BMI, HEI-2015, education, marital status, smoking, drinking, diabetes, hypertension, frequency of dental floss/device usage, and frequency of mouthwash usage. In the subgroup analysis, the model is not adjusted for the stratification variable itself. Abbreviations: OR, odds ratio; CI, confidence interval; RA, rheumatoid arthritis; PIR, poverty income ratio; BMI, body mass index; HEI-2015, Health Eating Index-2015.

Variable	Count	OR (95% CI)	P value	P for interaction
**Overall**	1544	0.127 (0.018, 0.915)	0.037	
**Gender**				0.115
Male	631	0.002 (0.000, 0.146)	0.004	
Female	913	0.289 (0.032, 3.097)	0.281	
**Age**				0.759
20-45 years	316	0.595 (0.022, 19.117)	0.754	
46-69 years	1228	0.062 (0.005, 0.910)	0.038	
**Race**				0.713
Mexican American	195	8.779 (0.000, 1228200.000)	0.705	
Other Hispanic	156	0.043 (0.000, 86509.000)	0.668	
Non-Hispanic White	676	0.078 (0.006, 1.071)	0.051	
Non-Hispanic Black	429	0.036 (0.000, 3.389)	0.142	
Other Races	88	0.172 (0.025, 1.211)	0.071	
**Education level**				0.05
Above high school	754	0.534 (0.040, 10.629)	0.651	
High school or equivalent	356	0.028 (0.000, 6.806)	0.179	
Under high school	432	0.002 (0.000, 0.205)	0.01	
**Marital status**				0.786
Married/Living with partner	876	0.118 (0.010, 1.370)	0.083	
Never married	187	1.802 (0.000, 1324600.000)	0.926	
Widowed/Divorced/Separated	479	0.127 (0.002, 7.268)	0.286	
**PIR**				0.021
<3.5	1024	0.055 (0.006, 0.479)	0.009	
≥3.5	404	44.785 (0.131, 52167.734)	0.248	
**BMI**				0.721
<25 kg/m²	298	0.250 (0.003, 23.774)	0.522	
25-30 kg/m²	430	0.047 (0.001, 1.665)	0.086	
≥30 kg/m²	799	0.277 (0.012, 8.333)	0.439	
**Dietary quality(HEI-2015)**				0.472
<60	1054	0.148 (0.018, 1.267)	0.074	
≥60	308	0.007 (0.000, 1.677)	0.07	
**Drinking status**				0.251
No	394	0.655 (0.019, 46.261)	0.827	
Yes	1072	0.041 (0.003, 0.499)	0.013	
**Smoking status**				0.567
Never	660	0.040 (0.002, 0.951)	0.042	
Ever	449	0.215 (0.002, 31.303)	0.534	
Current	435	0.343 (0.014, 11.169)	0.512	
**Hypertension**				0.889
No	545	0.111 (0.008, 1.490)	0.09	
Yes	999	0.050 (0.005, 0.558)	0.013	
**Diabetes**				0.257
No	888	0.042 (0.005, 0.369)	0.004	
Yes	656	0.183 (0.010, 4.042)	0.264	
**Frequency of dental floss/device usage**				0.761
Never	507	0.078 (0.003, 1.669)	0.105	
<1 per day	484	0.110 (0.002, 9.031)	0.292	
=1 per day	443	0.349 (0.010, 18.181)	0.567	
**Frequency of mouthwash usage**				0.955
Never	566	0.071 (0.004, 1.476)	0.074	
<1 per day	327	0.118 (0.002, 5.945)	0.277	
=1 per day	540	0.268 (0.004, 23.030)	0.544	

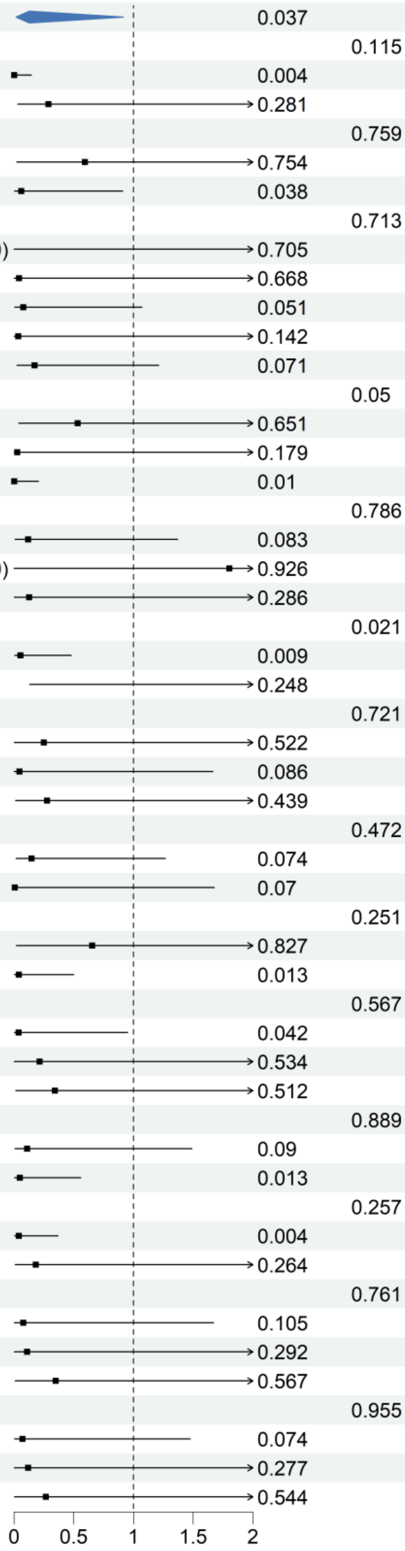

Supplement: Associations between oral microbiome diversity and rheumatoid arthritis in U.S. adults: NHANES 2009–2012 [file AOS-85-46064-s2.pdf]
